# Ecological and Landscape Factors Affecting the Spread of European Mistletoe (*Viscum album* L.) in Urban Areas (A Case Study of the Kaliningrad City, Russia)

**DOI:** 10.3390/plants9030394

**Published:** 2020-03-23

**Authors:** Liubov Skrypnik, Pavel Maslennikov, Pavel Feduraev, Artem Pungin, Nikolay Belov

**Affiliations:** 1 Institute of Living Systems, Immanuel Kant Baltic Federal University, Universitetskaya str., 2, Kaliningrad 236040, Russia; PMaslennikov@kantiana.ru (P.M.); PFeduraev@kantiana.ru (P.F.); APungin@kantiana.ru (A.P.); 2 Institute of Environmental Management, Urban Development and Spatial Planning, Immanuel Kant Baltic Federal University, Zoologicheskaya str., 2, Kaliningrad 236022, Russia; NBelov@kantiana.ru

**Keywords:** *Viscum album* L., mistletoe, distribution, urban ecology, plant parasite

## Abstract

Green spaces are very important for an urban environment. Trees in cities develop under more stressful conditions and are, therefore, more susceptible to parasite including mistletoe infestation. The aim of this study was to investigate the ecological, microclimatic, and landscape factors causing the spread of European mistletoe (*Viscum album* L.) in urban conditions. The most numerous hosts of mistletoe were *Tilia cordata* (24.4%), *Acer platanoides* (22.7%), and *Populus nigra* (16.7%). On average, there were more than 10 mistletoe bushes per tree. The mass mistletoe infestations (more than 50 bushes per the tree) were detected for *Populus × berolinensis*, *Populus nigra*, and *Acer saccharinum*. The largest number of infected trees was detected in the green zone (city parks), historical housing estates, and green zone along water bodies. Based on the results of principal component analysis (PCA), the main factors causing the spread of mistletoe on the urban territories are trees’ age and relative air humidity. The factors reflecting environmental pollution (the content of heavy metals in the soil and the concentration of nitrogen dioxide in the air) did not statistically affect the mistletoe distribution in the study area. However, this result may be due to the heterogeneity of other parameters in the studied areas. Therefore, additional research is required to more accurately interpret the data on the relationship between environmental pollutions and distribution of mistletoe infestation of trees in urban areas.

## 1. Introduction

Urban green spaces have been increasingly recognized as key components of urban planning [1]. The trees as urban keystone flora have a multitude of functions, such as to reduce air pollution, support biodiversity, mitigate heat island effects, increase land value, improve aesthetics, and even improve human health [2,3]. However, the urban environments present enormous challenges for tree growth, including elevated temperatures, water and nutrient constraints, and lack of adequate root and crown volumes [4]. Under the influence of adverse environmental conditions, urban trees become more sensitive to biotic stressors (e.g., hemiparasitic plants, herbivorous insects, or pathogens). The hemiparasitic plants that infest trees and cause particular concern are mistletoes, which have been determined as one of the increasing agents of forest decline worldwide [5].

Most mistletoes are an evergreen plant that develops stable haustorium in the host tree. The plants assimilate their own carbon due to photosynthesis, which determines its green color, while completely depending on the water and mineral resources of the tree on which it settles [6]. It is believed that mistletoes affect the yield of plantings, lead to a decrease in the growth energy of woody plants and their longevity, the loss of decorativeness and yield, and cause partial or continuous top drying and gradual drying of trees ultimately [7,8,9,10].

The mistletoe genus *Viscum* belongs to the mistletoe family *Santalaceae* (according to the APG (Angiosperm Phylogeny Group) IV system of flowering plant classification) and includes about 70 species of evergreen semiparasitic plants distributed in the subtropical and tropical zones of Africa, Madagascar, Asia, Australia, and the temperate zone of Eurasia [11]. 

Depending on the type of host tree, four subspecies of European mistletoe are distinguished, three of which are widely represented in Europe and the fourth subspecies was found only in Crete. *V. album* subsp. *album* L. grows on deciduous plants. *V. album* subsp. *abietis* (Wiesb.) grows on trees genus *Abies. V. album* subsp. *austriacum* (Wiesb.) is pine mistletoe that grows on pines and is less commonly found on fir trees. The fourth subspecies of *V. album* subsp. *creticum* N. Boehling et al. grows on *Pinus brutia* Ten. and is exclusive to Crete [6,12,13]. 

The natural southern and western borders of the range of European mistletoe are the Mediterranean Sea and the Atlantic Ocean. In the east, it is found in the Ukrainian Carpathians, as well as to some extent in other parts of western Ukraine and on the Crimean peninsula. In northern Europe, European mistletoe is sporadically found in Denmark and the southern regions of Sweden [14]. The northern border of the range of European mistletoe in the Baltic States runs through the territory of Latvia [15]. 

The spread of mistletoe is influenced by a whole range of biotic and abiotic factors. Among the biotic factors, the most important are the behavior of animals, especially birds, as the main agents carrying mistletoe seeds [16], the stand density of the mistletoe itself (intraspecific competition), and competition with other parasites [17], as well as the distribution and presence of suitable host trees and the physiological parasite–host relationships [18,19].

One of the most significant abiotic factors affecting the life of the mistletoe is temperature. Therefore, the optimum temperature for mistletoe ranges from 15 to 20 °C. Germination can also occur at lower temperatures, but takes much longer, 10 to 31 days, while at 18–32 °C takes 2 to 8 days in total [20]. The geographical distribution of mistletoe is limited by both summer and winter temperatures. The monthly average temperatures of the coldest and warmest months of the year correlate with the distribution limits of the *V. album*. Consequently, mistletoe is likely to benefit from climate warming and expand its range [14].

Other abiotic factors in general positively affecting the distribution of mistletoe are high soil moisture and air humidity, land use changes (increase in fragmented landscapes and narrow linear patches of vegetation) [21], and air pollution, especially by nitrogen compounds [22,23].

Previous work highlighted that mistletoe infection occurs more commonly in urban areas than in natural areas [24]. It is believed that urban trees are more likely to become infected with mistletoe due to the stressful conditions in which they are found. These trees grow on poor quality soils with a low content of nutrients and water, presence of pollutants, and high compaction due to continuous trampling and shallow depth [25,26]. In addition, across urban environments, heat islands with scarce vegetation cover are very common. Therefore, microclimatic conditions can rapidly change with subsequent changes in water and mineral balances. The need to study mistletoe in urban areas is, first of all, due to its rather widespread distribution in these territories. However, the works devoted to studies of the distribution of European mistletoe in cities depending on the degree of urbanization are insufficient and they are mainly presented by studying the distribution of mistletoe in cities in central and southwestern Poland (Warsaw, Lodz, and Praška) [20,22,27]. In addition, there is no research that has studied the complex effect of various factors of the urban environment on the distribution of mistletoe in urban areas.

The aim of this study was to investigate the distribution pattern of the European mistletoe in urban conditions, with reference to the city of Kaliningrad. In addition, we aimed to identify and discuss the main landscape and environmental factors affecting this distribution.

## 2. Results

### 2.1. Hosts’ Preference of European Mistletoe in the Reserch Area

In the investigated area of Kaliningrad, over 43,000 mistletoe specimens were recorded on 3661 host trees. Table 1 presents the species of trees on which mistletoe bushes were found. *V. album* was identified on 50 taxa belonging to 17 genera and nine families. As a result of studies, no infected conifers were identified, which indicates the distribution of the subspecies *Viscum album* L. subsp. *album* only in the city.

The most numerous hosts of mistletoe were Tilia cordata (24.4%), Acer platanoides (22.7%), Populus nigra (16.7%), Salix alba (6.3%), Acer saccharinum (4.3%), Crataegus monogyna (3.7%), Tilia platyphyllos (3.7%), Sorbus aucuparia (3.7%), Populus × berolinensis (2.6%), Betula pendula (1.9%), Malus domestica (1.9%), and Acer pseudoplatanus (1.4%). These 12 host species accounted for about 95 % of the total number of records. Most of the infected trees belonged to the families Malvaceae (genus Tilia, 29.4%), Sapindaceae (genus Acer, 28.5%), Salicaceae (genus Populus, 20.3%, and genus Salix, 6.4), and Rosacea (11.3%).

### 2.2. Intensity of Mistletoe Infestation Depending on Tree Species

Assessment of the mistletoe infection degree of trees showed that on average there were more than 10 mistletoe bushes per tree (Table 1). Depending on the number of bushes on one tree, low (less than 10 bushes), medium (10 to 30 bushes), high (30 to 50 bushes), and very high (more than 50 bushes) infection rates were distinguished. The first group included 62% of infected plants, the second group 29%, the third group 6%, and the last one about 2%. 

The results of the studies of the degree of the hosts’ crown infestation in relation to the host species are presented in Table 2. Despite the fact that the largest number of infected trees was detected among the species *Tilia cordata* and *Acer platanoides*, most of these trees were characterized by a low degree of mistletoe infection. The species of host trees on which the foci of mass mistletoe were detected (more than 50 bushes on the tree) included, first of all, *Populus × berolinensis*, *Populus nigra*, and *Acer saccharinum*. More than 5% of the trees of these species belonged to the fourth group, a “very high” degree of infection.

### 2.3. Distribution of Mistletoe Infestation under the Urban Conditions

**Figure 1:**
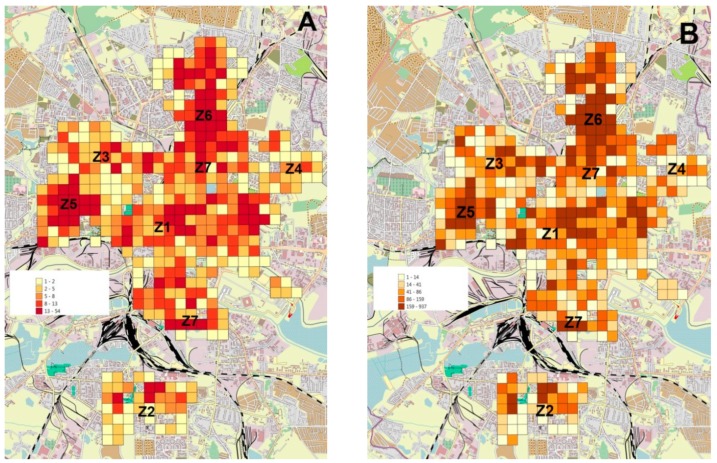
The map of mistletoe infestation occurrence in the city of Kaliningrad. (**A**) The frequency of occurrence of host trees attacked by *V. album* subsp. *album* in the research area. (**B**) The frequency of occurrence of *V. album* subsp. *album* in the research area. The labels Z1‒Z7 indicate the centers of research zones according to Table 3.

At the first stage of the study, a general assessment of the geographical distribution of mistletoe in the city of Kaliningrad was performed. Geographical distribution analysis of mistletoe-infected trees in the city showed that the main distribution areas are mosaic and located inconsistently (Figure 1A,B). A comparison of the results presented in Figure 1A,B shows that the areas with the maximum number of mistletoe-infected trees almost completely coincided with the areas in which the number of mistletoe bushes was also high. This result indicates that the average infection rate (the number of mistletoe bushes per tree) was independent of the mistletoe distribution areas.

In the next phase of research to identify the main ecological and landscape factors affecting the mistletoe distribution in the city, within the research area, seven zones differing in the degree of anthropogenic load were selected. For these zones, some landscape, microclimatic, and environmental parameters were investigated. The results are shown in Table 3.

The studied zones differed significantly in the presence of mistletoe-infected trees in their territory. The largest number of infected trees was detected in the green zone (city parks), historical housing estates, and green zone along the water bodies. The zone of modern multistory apartments was characterized by the lowest degree of infestation. The city center, zone of medium-rise apartments, and zone of private residential buildings were characterized by an average degree of infestation. The diameter of mistletoe bushes in the studied zones varied widely from 20 to 180 cm, but averaged from 45 to 75 cm. 

The presence of a higher density of roads and buildings in the city center and in the zone of modern multistory apartment block estates was accompanied by a higher average air temperature and lower relative humidity. The city center was also characterized by a higher concentration of nitrogen dioxide in the air. However, the content of lead and zinc in the soil was higher in zone Z3 (medium-rise apartment block estates).

**Figure 2:**
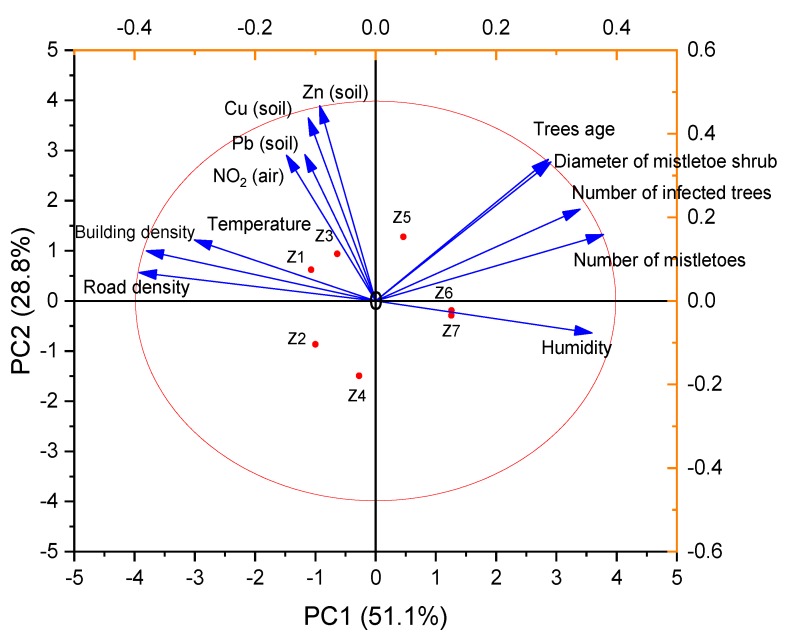
Principal component analysis (PCA) biplot of the concatenated data exhibiting the correlation between mistletoe occurrence parameters and landscape, climatic, and environmental factors.

The results of a multivariate statistical analysis of the obtained experimental data are presented in Figure 2 in the form of a principal component analysis (PCA) biplot.

The total of six principle components can explain 100% of the total variance. The first principal component (PC1) represented 51.1% of the variance, and the second principal component (PC2) represented 28.8 % of the variance. The next two principal components represented 7.95% (PC3) and 6.21% (PC4) of the variance, respectively. The 95% bootstrapped confidence interval for PC1 was 41.9–79.3 % (N = 999). The performed PCA revealed the relationship between the age of the trees, humidity, and parameters of mistletoe infestation. The closest connection was established between the age of the trees and the diameter of the mistletoe bushes. The amount of mistletoe increased with increasing relative humidity in the area studied. However, between the parameters of mistletoe distribution and landscape factors (the density of the road network and the density of buildings), an inverse correlation was revealed. The areas studied that were characterized by a higher local temperature, had a lower level of mistletoe infestation. No correlation was found between the factors reflecting environmental pollution (the content of heavy metals in the soil and the concentration of nitrogen dioxide in the air) and the parameters of mistletoe distribution.

## 3. Discussion

Despite the accumulated experimental material on species susceptible to mistletoe infestation, the strategy for choosing a host tree by mistletoe is still unclear. For example, the in the paper by Barney et al. [28] a list of 452 taxa, on which European mistletoe was identified in Europe, Asia, and California, was presented. Moreover, it is possible that the preferences of mistletoe also depend on the geographical region of its growth and the characteristics of local populations. Linden, maple, and poplar trees predominated among the recorded infected tree species in this study. These species are often used in urban woodland, especially along roads. The strong susceptibility of linden trees to mistletoe infestation was also established earlier for some European cities. However, this species was not recorded among the most infected in a number of studies on the distribution of European mistletoe in cities in Poland bordering the Kaliningrad region [22,27]. In addition, in the same studies, mistletoe was rarely found on *Acer platanoides*, which was severely affected by mistletoe in Kaliningrad. It is believed that European mistletoe prefers trees with soft wood [14]. However, the significant frequency of its occurrence on maple, belonging to solid species of trees, suggests that it is not possible to single out one factor that determines the choice of host tree by mistletoe. In addition, a study of the distribution of mistletoe in the territory of the Botanical Garden showed that introduced tree species were more susceptible to mistletoe infestation. Among the infected tree species in the territory of the Botanical Garden were species such as *Styphnolobium japonicum*, *Juglans mandshurica*, and *Aesculus glabra*. The number of infected Acer saccharinum trees was 5 times lower compared to the number of infected. However, the result could be explained with a generally low percentage of this species in the wooded area of the observed city, as almost 100% of *Acer saccharinum* was proven to be infected. A strong susceptibility of *Acer saccharinum* to mistletoe infestation has also been reported in previous studies [14,22,27]. In this regard, one of the measures to reduce the distribution of mistletoe is to plant the trees that are less likely to be attacked by mistletoe (for example, oaks, hornbeams, and beech trees). 

The largest number of infected trees was detected in the green areas (city parks), historical housing estates, and green zone along the natural or artificial lakes, ponds, and waterways. Similar results were obtained in a study of the mistletoe distribution in Warsaw [27]. At the same time, as well as for Warsaw, Kaliningrad was not characterized by the presence of mistletoe in urban forests, in which trees infected with mistletoe were found only at the outer borders. As noted by several authors, an adequate amount of sunlight is an important condition for the mistletoe distribution [29,30]. For the same reason, some authors believe that thinning trees will contribute to the mistletoe distribution [31,32]. The city center, featuring a higher road and construction density, was observed to have a relatively low level of mistletoe distribution. This could be associated not only with its generally low concentration of “green” areas, but also with a better and more regular maintenance of old and damaged trees.

A higher level of mistletoe-infected trees in the historical housing estates was due to the prevalence of older trees there. The age of the tree is also a factor in the mistletoe distribution. Despite the fact that mistletoe can also affect young trees, in most studies there was reported a direct correlation between the mean age of trees in green spaces and the level of mistletoe infestation [14,27,33].

The presence of a direct correlation between the degree of mistletoe infestation and air humidity, as well as the predominance of mistletoe-affected trees in green areas along water bodies, can be due to several reasons. First, tree species that have an increased water balance and hygrophytes are most often susceptible to infection. The moisture content is a prerequisite for both the germination of mistletoe seeds, and for its successful development. At the first stages of ontogenesis, when the mistletoe is just beginning to damage the tree, the moisture in the air is an additional source of water for it. Secondly, natural and artificial reservoirs and streams in cities represent a rich forage base for various species of birds, which are the main carriers of mistletoe seeds and contribute to its distribution. The most important birds for mistletoe dispersal are the mistle thrush (*Turdus viscivorus*), fieldfare (*Turdus pilaris*), waxwing (*Bombycilla garrula*), and blackcap (*Sylvia atricapilla*) [6]. According to the Atlas of Breeding Birds of Kaliningrad [34], *Turdus pilaris* and *Sylvia atricapilla* nest in the city of Kaliningrad. The highest nesting density of *Sylvia atricapilla* was recorded in the western part of the city, including in the historical residential area, where a high level of mistletoe infestation in trees was revealed. *Bombycilla garrula* do not nest in the research area, but are represented by a rather large population in the city and, according to bird watchers, are the main distributors of mistletoe.

According to available literature data, one of the most important factors determining the mistletoe distribution is temperature. It is believed that increasing average annual temperatures and climate change contribute to the mistletoe distribution in new areas [35]. However, in our study, an inverse correlation was found between temperature and mistletoe distribution parameters. Such a contradictory result is probably due to the fact that higher temperatures in the area studied were set in the center of the city, which, as mentioned above, was characterized by a smaller share of tree plantings and more careful care for them. Another possible reason for the lower mistletoe infestation in areas with higher temperature is lower relative humidity detected in these zones. For example, previous studies in the city of Kaliningrad showed the negative effect of reducing air humidity in the city center, as a result of heat island formation, on the urban flora especially on biodiversity of epiphytic lichens [36]. The result obtained in the presented study indicates that insufficient moisture is the main factor limiting the mistletoe distribution in an urban environment. In the study by Molnár and Végvári [37], it was shown that dryness in July was the main factor limiting the mistletoe spread, followed only by average July temperature and amount of air temperature during the growing period.

Despite the fact that some authors point to the relationship between the level of nitrogen dioxide in the air and the degree of mistletoe infestation of trees [22], no correlation between these parameters was established in this study. The result obtained may be due to the heterogeneity of other parameters in the studied areas (density of the road network, building density, humidity, presence of birds, accessibility of host trees, etc.). Therefore, additional research is required to more accurately interpret the data obtained. This conclusion also refers to the results indicating the absence of a correlation between the content of heavy metals in the soil and the parameters of the mistletoe infestation of trees. The study by Sharm et al. indicated that in areas with a higher content of heavy metals in the soil, mistletoe infestation of trees was also higher [38]. However, as the authors themselves noted, the effect of heavy metals on the mistletoe distribution was indirect and was primarily associated with the deterioration of the physiological state of trees under their influence, which complicated the unambiguous interpretation of the data.

## 4. Materials and Methods 

### 4.1. Research Area

**Figure 3:**
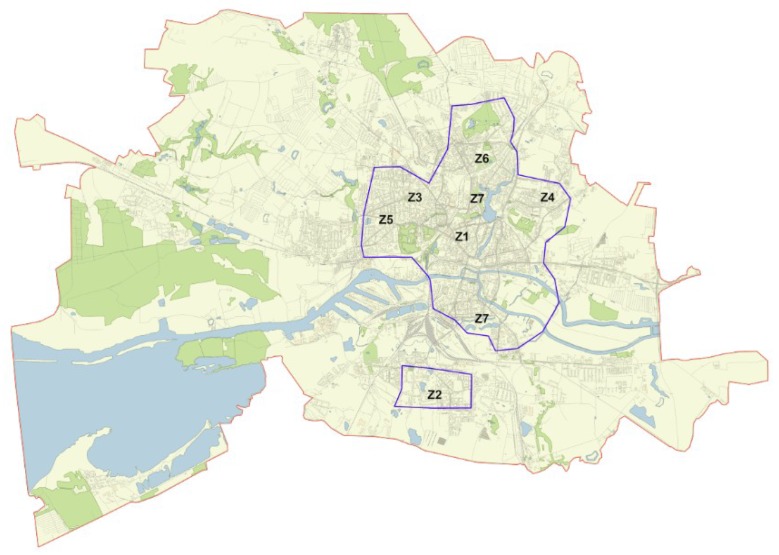
The map of the city Kaliningrad (red line), research area (blue line), and the research zones (Z1–Z7).

A study of the distribution of mistletoe and identification of trees affected by it was carried out in the city of Kaliningrad (54°43′ N, 20°30′ E) from April to December 2019. Kaliningrad is the administrative center of the Kaliningrad region, located in the western part of Russia on the Baltic coast. From the south, the Kaliningrad region borders Poland, and in the north and east, Lithuania. Kaliningrad is located in the Primorsk Lowland, on the banks of the Pregolya River at its confluence with the Kaliningrad (Vistula) Bay. The climate of Kaliningrad is transitional from temperate to temperate continental. Air temperature in winter is –5 to 5 °C and in summer is 18 to 23 °C. The warmest month of the year is July, with an average daily temperature of 18.1 °C. The coldest is January, with an average temperature of –1.5 °C. The average annual temperature is 7.9 °C. The average annual rainfall is 804 mm (1981–2010). The total area of the city is 223 km^2^. The population is 482,400 people (as of 1 January 1 2019). The total area of the territory studied was 25 km^2^ and included the central part of the city (Figure 3).

### 4.2. Distribution of Mistletoe Infestation in the City

Due to the fact that the spread of mistletoe was not previously studied in the city of Kaliningrad, at the first stage of the presented study, the general distribution of mistletoe was explored. For this purpose, in the area shown in Figure 3 all mistletoe-infected trees were identified. For each infected tree the following information was recorded: Coordinates, species, tree diameter (at a height at 1.3 m), number of mistletoe bushes on tree, and average diameter of the mistletoe bushes (the minimum and maximum diameter was also recorded in the case of heterogeneity of sizes). Scientific species’ names and species’ authorities for plants were given according to the World Flora Online (http://www.worldfloraonline.org/) [39].

Four categories were distinguished according to the degree of damage to the crown of mistletoe trees: Low (less than 10 bushes per tree), medium (10 to 30 bushes), high (30 to 50 bushes on a tree), and very high (more than 50 bushes per tree). The classification proposed by the authors was based on the Barbu [40] rating system of mistletoe infestation intensity, which takes into account the site of mistletoe damage (lateral branches, crown, stem) and the percentage of the affected crown, as well as the presence and amount of dried branches infested with mistletoe.

To calculate the spatial patterns of mistletoe distribution and identify relationships, QGIS 3.6 with the Spreadsheet Layers module was used. The SasPlanet software package (stable version v. 190707) was used to obtain a raster substrate for the studied areas. In order to increase the accuracy of estimating the European mistletoe distribution, the method of zonal statistics with elements of focal statistics was chosen. The size of the computational cell was set to 250 m*250 m (6.25 ha), which is most optimal for cities of such a size as Kaliningrad. Using GIS, the total number of affected trees and total number of mistletoe bushes, as well as road and building density for each studied square, was determined.

### 4.3. Distribution of Mistletoe Infestation in Zones Differed by Anthropogenic Load

In order to assess the features of the spread of mistletoe depending on the anthropogenic load in the surveyed area, seven zones were selected. These zones differed in a certain type of habitat, from seminatural to the most altered as a result of human activity. The allocation of zones was carried out on the basis of the master plan of the city of Kaliningrad. The size of each zone was 1 km^2^ (100 ha). A brief description of each zone is presented in Table 4. 

In addition to the assessment of the level of mistletoe distribution, the following parameters were determined for each zone: Microclimatic features (temperature, humidity), soil contamination with heavy metals (average content of lead, zinc, and copper in soil), and atmospheric air pollution with nitrogen dioxide.

### 4.4. Climatic Parameters and Enviromental Pollutants Measuring

Temperature and humidity in the areas studied were determined using portable sensors (UNI-T UT330C USB). For this purpose, three trees were selected in each of the seven zones, on which sensors were mounted on the south side at a height of 3 meters from the ground. Temperature and humidity were recorded around the clock during the first week of April, July, and December 2019. Then the data on temperature and humidity were averaged for each zone and used for statistical analysis.

The metal content in soil samples taken from the territories of the studied zones was determined by X-ray fluorescence analysis on a Spectroscan Max-G instrument (NPO Spectron LLC, Russia). Then, 5–6 samples were taken from each zone, which were then combined into a mixed sample and used for analysis. Sample preparation and analysis were carried out according to [41].

Measurements of nitrogen dioxide in the air of the research zones were carried out by using a passive sampling method with spectrophotometric determination as described in [42].

### 4.5. Statistical Analysis

The statistical analysis of the obtained experimental data was performed using OriginPro 2019b (OriginLab Corporation, Northampton, MA, USA). To evaluate the relationship between the parameters of mistletoe infestation (number of infected trees, number of mistletoe specimens, diameter of mistletoe shrub) and ecological and landscape factors, the principal component analysis (PCA) was used. The assessment of loadings’ significance in PCA was performed using the bootstrap (N = 999) according to Peres-Neto et al. [43]. The 95% bootstrapped confidence intervals for the eigenvalues were evaluated using Past v. 4.01. One–way analysis of variance (ANOVA) and Kruskal–Wallis tests were used for comparing the research zones. Difference among means was determined by Tukey’s test at a significance level of *p* ≤ 0.05. 

## 5. Conclusions

Performed for the first time, a study of the spread of mistletoe in the city of Kaliningrad revealed that the main tree species susceptible to mistletoe infestation were *Tilia cordata* (24.4 %), *Acer platanoides* (22.7 %), and *Populus nigra* (16.7%). In addition, it was found that most of *Acer saccharinum* trees in Kaliningrad are infested by mistletoe, which would allow us to recommend excluding it from urban plantings and favoring more resistant species such as oaks, hornbeams, and beech trees. An analysis of the environmental and landscape factors causing mistletoe spreading under urban conditions (with reference to the city of Kaliningrad) showed that the main factors that positively affected the spread of mistletoe were the age of the trees and the relative humidity. The factors reflecting environmental pollution (the content of heavy metals in the soil and the concentration of nitrogen dioxide in the air) did not statistically affect the mistletoe distribution in the study area. However, additional research is required to more accurately interpret the data on the relationship between environmental pollutions and distribution of mistletoe infestation of trees in urban areas.

## Figures and Tables

**Table 1:** Hosts of European mistletoe in the city of Kaliningrad ^1^.

Tree Scientific Name	Number of Infected Trees	Number of Mistletoe Specimens	Rate of Mistletoe Infestation ^2^
*Tilia cordata* Mill.	894	9925	11.1
*Acer platanoides* L.	831	6554	7.9
*Populus nigra* L.	613	11032	18.1
*Salix alba* L.	229	3509	15.3
*Acer saccharinum* L.	157	3155	20.1
*Crataegus monogyna* Jacq.	137	1194	8.7
*Tilia platyphyllos* Scop.	137	1432	10.5
*Sorbus aucuparia* L.	134	1081	8.1
*Populus × berolinensis* K.Koch.	96	1912	19.9
*Betula pendula* Roth	69	335	4.9
*Malus domestica* Borkh.	68	448	6.6
*Acer pseudoplatanus* L.	51	349	6.8
*Robinia pseudoacacia* L.	30	206	6.9
*Fraxinus excelsior* L.	27	214	7.9
*Tilia begoniifolia ‘Euchlora’* Steven	24	201	8.4
*Crataegus laevigata* (Poir.) DC.	30	213	7.1
*Populus balsamifera* L.	20	251	12.6
*Malus baccata* (L.) Borkh.	14	246	17.6
*Tilia tomentosa* Moench	11	92	8.4
*Crataegus chlorosarca* Maxim.	8	30	3.8
*Populus tremula* L.	8	83	10.4
*Betula papyrifera* Marshall	7	58	8.3
*Tilia americana* L.	7	42	6.0
*Populus laurifolia* Ledeb.	6	36	6.0
*Crataegus sanguinea* Pall.	5	33	6.6
*Malus × prunifolia* (Willd.) Borkh.	5	69	13.8
*Prunus cerasifera* Ehrh.	5	24	4.8
*Carpinus betulus* L.	4	18	4.5
*Salix caprea* L.	4	19	4.8
*Aesculus glabra* Willd.	3	21	7.0
*Prunus padus* L.	3	33	11.0
*Acer negundo* L.	2	4	2.0
*Betula pubescens* Ehrh.	2	28	14.0
*Populus x canadensis* Moench	2	50	25.0
*Quercus rubra* L.	2	4	2.0
*Sorbus intermedia* (Ehrh.) Pers.	2	8	4.0
*Tilia caroliniana* Mill.	2	43	21.5
*Acer hyrcanum* Fisch. & C.A.Mey.	1	20	20.0
*Alnus glutinosa* (L.) Gaertn.	1	12	12.0
*Betula dahurica* Pall.	1	4	4.0
*Betula lenta* L.	1	4	4.0
*Juglans mandshurica* Maxim.	1	6	6.0
*Malus niedzwetzkyana* Dieck ex Koehne	1	4	4.0
*Malus pumila ‘Elise Rathke’* Mill.	1	4	4.0
*Populus simonii* Carrière	1	3	3.0
*Prunus domestica* L.	1	3	3.0
*Quercus robur* L.	1	12	12.0
*Salix triandra* L.	1	2	2.0
*Styphnolobium japonicum* (L.) Schott	1	6	6.0
**Total**	**3661**	**43032**	**11.8**

^1^ The data are organized considering the number of infested specimens of trees species. ^2^ The rate of mistletoe infestation presents the number of mistletoe bushes per tree.

**Table 2:** Distribution of various tree species according to the degree of infection with mistletoe.

Host Species ^1^	Number of Host Trees (% to Total Tree Number of This Specie)			
	Low	Medium	High	Very High
*Tilia cordata*	549 (61.5)	284 (31.7)	57 (6.4)	4 (0.4)
*Acer platanoides*	628 (75.6)	180 (21.7)	22 (2.6)	1 (0.1)
*Populus nigra*	271 (44.2)	233 (38.0)	70 (11.4)	39 (6.4)
*Salix alba*	125 (54.6)	77 (33.6)	19 (8.3)	8 (3.5)
*Acer saccharinum*	65 (41.4)	55 (35.0)	28 (17.8)	9 (5.7)
*Crataegus monogyna*	96 (70.1)	40 (29.2)	1 (0.7)	-
*Tilia platyphyllos*	88 (64.2)	44 (32.1)	5 (3.6)	-
*Sorbus aucuparia*	96 (71.6)	36 (26.9)	2 (1.5)	-
*Populus × berolinensis*	35 (36.5)	41 (42.7)	13 (13.5)	7 (7.3)
*Betula pendula*	60 (87.0)	8 (11.6)	1 (1.4)	-
*Malus domestica*	52 (76.5)	16 (23.5)	-	-
*Acer pseudoplatanus*	42 (82.4)	7 (13.7)	1 (2.0)	1 (2.0)

^1^ Data are presented for 12 species of trees, characterized by a significant number of infected trees (a total of more than 90%).

**Table 3:** The occurrence of mistletoe infestation and landscape, ecological, and microclimatic features of research zones.

Parameters	Research Zones ^1^						
	Z1	Z2	Z3	Z4	Z5	Z6	Z7
The Total Number of Trees (Per Zone)	1811	1268	2243	1346	2145	2456	1700
Number of infected trees (per 1 km^2^) (Percent to the total number of trees, %)	192b ^2^ (10.6)	64d (5.0)	176bc (7.8)	128cd (9.5)	384a (17.9)	464a (18.9)	304a (17.8)
Number of mistletoes (per 1 km^2^)	1392cd	592e	1872c	1008de	5424b	6848a	5616ab
Medium diameter of mistletoe shrub, cm	60bc	55cd	65bc	45d	75ab	75ab	75ab
Medium trees age, years	50cd	30e	60bc	40de	80a	80a	60bc
Road density, 10^3^ km per 1 km^2^	18.5b	20.2a	18.5b	13.2c	11.9c	5.3d	2.7d
Building density, 10^3^ m^2^ per 1 km^2^	262.9a	181.5b	156.6b	107.7c	104.7c	20.4d	6.9d
Temperature, °C	9.5a	8.9ba	9.4a	8.6b	8.4b	8.8b	7.6c
Relative humidity, %	70.2c	69.3c	74.1bc	76.2b	74.7bc	78.3ab	83.3a
Concentration of NO_2_, µg m^-3^	32a	24bc	23bc	17d	27ab	19cd	24b
Pb content in soil, mg kg^-1^	73bc	38de	142a	22e	121a	23e	49cd
Zn content in soil, mg kg^-1^	152b	104c	243a	64d	112c	97c	131b
Cu content in soil, mg kg^-1^	48b	15c	42b	18c	72a	8d	12cd

^1^ Z1, city center; Z2, modern multistory apartment block estates; Z3, medium-rise apartment block estates; Z4, single-family households with gardens; Z5, historical housing estates; Z6, green areas (city parks); Z7, green areas along natural or artificial lakes, ponds, and waterways. ^2^ Different letters indicate significantly different means according to Tukey’s HSD (honestly significant difference) test (n = 16, *p* ≤ 0.05).

**Table 4:** Description of seven studied zones differing by the level of human impact on the territory.

Zone Abbreviation	Zone Name	Description
Z1	City center	The mixed type zone, including a complex of administrative buildings, shopping centers, educational institutions, etc., the main highways of the city, as well as recreational green spaces and small parks
Z2	Modern multistory apartments blocks estates	Modern residential buildings with open courtyards, mostly covered with lawns, have a low proportion of woody vegetation.
Z3	Medium-rise apartments blocks estates	Residential apartment buildings are mainly 5–8 story, buildings of the second half of the twentieth century with open courtyards, separate green areas and trees along the roads
Z4	Single-family households with gardens	Single-family residential buildings with gardens
Z5	Historical housing estates	A complex of old low-rise houses built in the early 20th century with private courtyards and gardens having well-developed green areas with old trees
Z6	Green areas (city parks)	Extensive areas of higher vegetation, such as parks, garden plots, squares
Z7	Green areas along natural or artificial lakes, ponds, and waterways	Green belts along natural or artificial lakes, ponds, and waterways in the city
